# Hi-Enhancer: a two-stage framework for prediction and localization of enhancers based on Blending-KAN and Stacking-Auto models

**DOI:** 10.1093/bioinformatics/btaf441

**Published:** 2025-12-08

**Authors:** Aimin Li, Haotian Zhou, Rong Fei, Juntao Zou, Xiguo Yuan, Yajun Liu, Saurav Mallik, Xinhong Hei, Lei Wang

**Affiliations:** Shaanxi Key Laboratory for Network Computing and Security Technology, Xi’an University of Technology, Xi’an, Shaanxi 710048, China; Shaanxi Key Laboratory for Network Computing and Security Technology, Xi’an University of Technology, Xi’an, Shaanxi 710048, China; Shaanxi Key Laboratory for Network Computing and Security Technology, Xi’an University of Technology, Xi’an, Shaanxi 710048, China; Department of Materials Science and Engineering, Xi’an University of Technology, Xi’an, Shaanxi 710048, China; School of Computer Science and Technology, Xidian University, Xi’an, Shaanxi 710071, China; Shaanxi Key Laboratory for Network Computing and Security Technology, Xi’an University of Technology, Xi’an, Shaanxi 710048, China; Department of Environmental Health, Harvard University T.H. Chan School of Public Health, Boston, MA 02115, United States; Shaanxi Key Laboratory for Network Computing and Security Technology, Xi’an University of Technology, Xi’an, Shaanxi 710048, China; Shaanxi Key Laboratory for Network Computing and Security Technology, Xi’an University of Technology, Xi’an, Shaanxi 710048, China

## Abstract

**Motivation:**

Gene expression plays a crucial role in cell function, and enhancers can regulate gene expression precisely. Therefore, accurate prediction of enhancers is particularly critical. However, existing prediction methods have low accuracy or rely on fixed multiple epigenetic signals, which may not always be available.

**Results:**

We propose a two-stage framework that accurately predicts enhancers by flexibly combining multiple epigenetic signals. In the first stage, we designed a Blending-KAN model, which integrates the results of various base classifiers and employs Kolmogorov–Arnold Networks (KAN) as a meta-classifier to predict enhancers based on flexible combinations of multiple epigenetic signals. In the second stage, we developed a Stacking-Auto model, which extracted sequence features using DNABERT-2 and located the enhancers based on the Stacking strategy and AutoGluon framework. The accuracy of the Blending-KAN model reached 99.69 ± 0.11% when five epigenetic signals were used. In cross-cell line prediction, the accuracy was more significant than or equal to 93.72%. With Gaussian noise, it still maintains an accuracy of 98.74 ± 0.03%. In the second stage, the accuracy of the Stacking-Auto model is 80.50%, which is better than the existing 17 methods. The results show that our models can be flexibly used to predict and locate enhancers utilizing a combination of multiple epigenetic signals.

**Availability and implementation:**

The source code is available at https://github.com/emanlee/Hi-Enhancer and https://doi.org/10.6084/m9.figshare.29262158.v1.

## 1 Introduction

The precise regulation of gene expression is essential for maintaining cellular function and organismal development. Regulatory elements such as promoters and enhancers, along with regulatory proteins like transcription factors (TFs), form this sophisticated regulatory network (Cramer 2019). Enhancers are a special class of DNA sequences that can remotely regulate the transcription of genes and enhance or repress gene expression by interacting with promoters (Dao and Spicuglia 2018).

Although the role of enhancers in transcriptional regulation is widely recognized, it remains a challenge to predict enhancers accurately. Traditional methods for enhancer identification mainly rely on biological experimental techniques, which are usually time-consuming, costly, and complicated to operate (Boyle *et al.* 2014). In recent years, machine learning and deep learning-based methods have become essential tools for enhancer prediction. These methods can predict enhancers and their functional properties from a large amount of genomic data by integrating multiple data types (Ernst and Kellis 2012), such as evolutionary conservation, epigenetic markers, DNA sequence motifs, and transcription factor binding sites. EnhancerFinder integrates DNA sequence motifs, evolutionary patterns, and functional genomic datasets of different cell types using a multinuclear learning approach to improve enhancer recognition (Erwin *et al.* 2014). iEnhancer-BERT is based on a pre-trained DNA language model and fine-tuned on the enhancer recognition task through a transfer learning strategy to extract deeper sequence features (Luo *et al.* 2022). iEnhancer-DHF combines Pseudo k-Tuple Nucleotide Composition (PseKNC) and FastText methods for feature extraction and the Deep Neural Network (DNN) model for enhancer prediction (Inayat *et al.* 2021). DECODE utilizes five kinds of signals (DNase-seq as well as ChIP-seq data of H3K27ac, H3K4me3, H3K4me1, and H3K9ac), to train a deep neural network for accurately predicting cell type-specific enhancers (Chen *et al.* 2021). In addition, DECODE implements a weakly supervised target detection framework for pinpointing enhancer boundaries.

Among all the above methods, DECODE has the highest accuracy in enhancer prediction. However, DECODE relies on five kinds of signals, which may not always be available.

This study aims to develop a new computational framework that can effectively predict and localize enhancers in a scenario where not all the above five kinds of signals are available. The framework developed here fuses several models and techniques. A concise description of how they work and their roles is provided in Text S1, available as supplementary data at *Bioinformatics* online. Similar to DECODE, our new framework, Hi-Enhancer, has two stages: (i) predicting whether an enhancer exists within a genomic region and (ii) if an enhancer exists, further localizing the boundaries of the enhancer.

In the first stage, we employed a model fusion technique to design a Blending-KAN model based on the AutoGluon framework (Erickson *et al.* 2020) and the KAN model (Liu *et al.* 2024) for determining whether an enhancer is contained in a genomic region. Blending-KAN integrates 123 base classifiers through Blending (Breiman 1996). We can efficiently identify the presence or absence of enhancers in genomic regions. Blending-KAN supports the flexible combination of multiple signals. It allows users to input one or more signals and obtains higher accuracy than DECODE.

In the second stage, we segmented the genomic regions containing enhancers and developed the Stacking-Auto model to localize the boundaries of enhancers. We used a sliding window to slice DNA sequences into 200 bp bins and extracted key features using DNABERT-2 (Zhou *et al.* 2023). Next, we designed the Stacking-Auto model to predict probabilities for each bin as an enhancer. Finally, based on these predicted probability values, we utilized a dynamic thresholding algorithm to pinpoint the complete boundaries of enhancers. The Stacking-Auto model combines the Stacking method (Wolpert 1992) with the AutoGluon framework(Erickson *et al.* 2020) to improve the model’s generalization performance, resulting in better performance than the existing 17 methods.

## 2 Materials and methods

### 2.1 Blending-KAN for predicting enhancer regions

#### 2.1.1 Preprocessing datasets

We collected various data of the human cell lines HCT116 and A549 from the ENCODE data portal (https://www.encodeproject.org/). The data collected include STARR-seq, chromatin accessibility (DNase-seq), and ChIP-seq for H3K27ac, H3K4me3, H3K4me1, and H3K9ac (Feingold *et al.* 2004). The rationale for selecting the two cell lines and a brief description of them are provided in Text S2, available as supplementary data at *Bioinformatics* online. We identified the overlap regions between the DNase-seq and STARR-seq peaks. If these overlapping regions intersect with any of the ChIP-seq peaks (H3K27ac, H3K4me3, H3K4me1, or H3K9ac), they are considered enhancer regions (positive samples) (Zhang *et al.* 2008). Negative samples (non-enhancer regions) were randomly selected from regions other than enhancers. The length of both positive and negative samples is 4000 bp, and the number ratio is 1:10 (see Text S3, available as supplementary data at *Bioinformatics* online). Finally, we obtained 8350 positive and 83 500 negative samples for model construction on the human cell line HCT116. For cross-cell line prediction, 3403 positive and 34 030 negative samples were obtained on the human cell line HA549. DNase-seq and ChIP-seq signals were aggregated in each 10 bp bin and averaged. The length of the signal data for each sample was 400 after aggregation. Table S1, available as supplementary data at *Bioinformatics* online provides a detailed overview of the Blending-KAN dataset.

#### 2.1.2 Blending-KAN model

**Figure 1.**
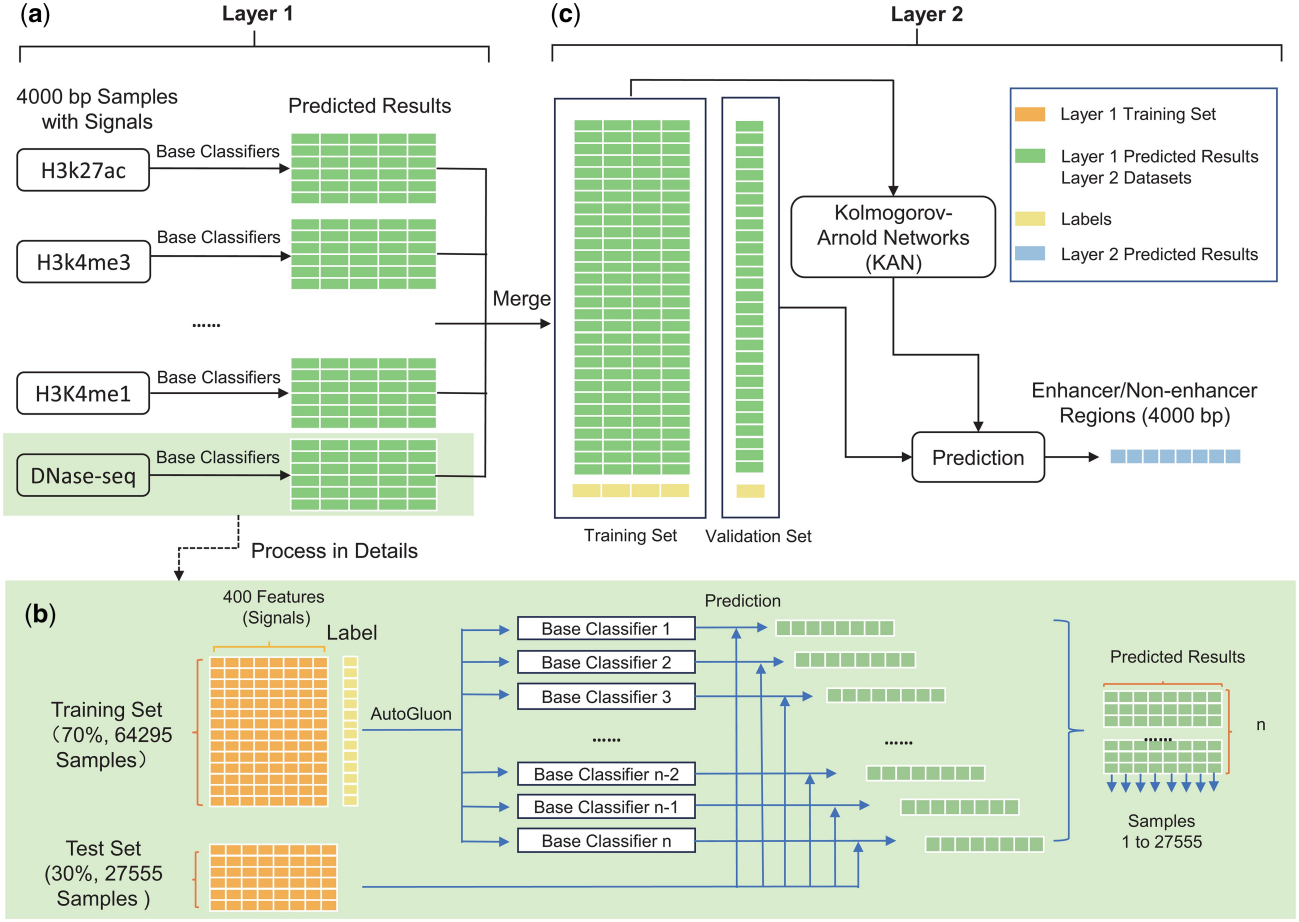
The architecture of the Blending-KAN model. (a) Layer 1 of Blending-KAN. (b) Details of layer 1. (c) Layer 2 of Blending-KAN.

Figure 1 illustrates the architecture of the Blending-KAN model, which uses Blending Learning (Breiman 1996). Our model supports flexible combinations of different kinds of signals (DNase-seq, H3K27ac, H3K4me3, H3K4me1, and H3K9ac). Base classifiers were trained via AutoGluon using the training set and then used to make predictions on the test set to generate a new set of prediction results. These predictions were input features for the KAN meta-classifier (Liu *et al.* 2024). Our model is described in detail as follows.

##### 2.1.2.1 *Layer 1 of Blending-KAN*

**Figure 2.**
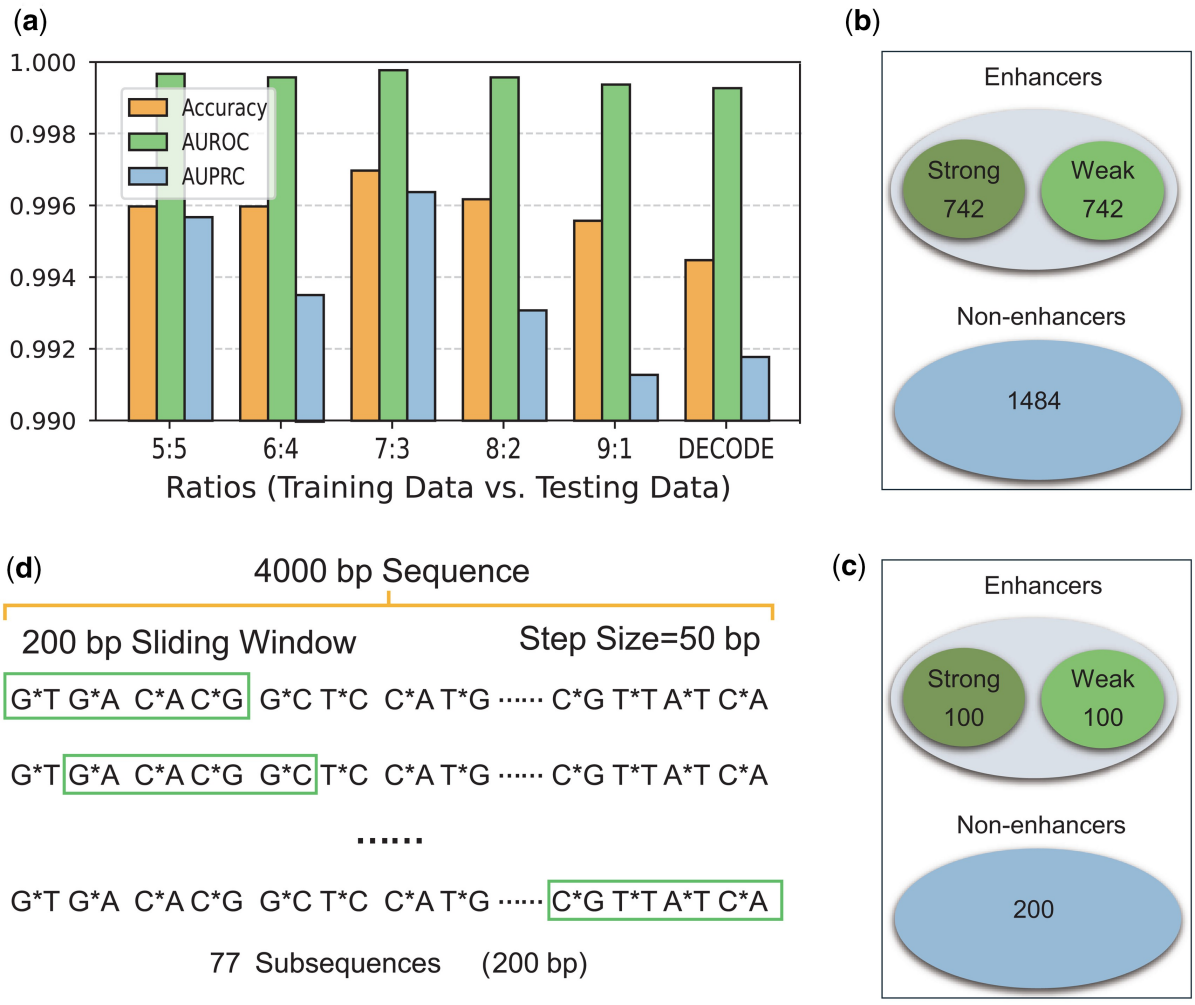
Datasets and processing. (a) Effect of data division ratio on the performance of Blending-KAN model. (b) Enhancers and non-enhancers in the training dataset. (c) Enhancers and non-enhancers in the testing dataset. (d) Sliding window to extract subsequences. “G*T” represents a sequence that starts with “G” and ends with “T” and whose length is 50 base pairs (bp).

The 4000 bp samples (including enhancer and non-enhancer regions) were divided into training and validation sets with a ratio of 7:3 (see Text S4, available as supplementary data at *Bioinformatics* online and Fig. 2a). We trained multiple base classifiers using AutoGluon, which use chromatin accessibility signals (DNase-seq) and epigenetic signals (such as H3K27ac) as input features. The task of the base classifiers was to preliminarily classify genomic regions based on the input epigenetic signals to determine whether the region was likely to contain enhancers. The prediction results of each base classifier on the validation set will be used as new features to construct a new training set. These new training sets will be used as input features for the second layer (Layer 2) for further model training and optimization. During the training process of the Layer 2 model, a five-fold cross-validation method is used, i.e. 20% of the data from the new training set is randomly selected as an independent test set each time to ensure the generalization ability and stability of the model.

To address the class imbalance problem, we assigned weights to the positive and negative samples, with the weight ratio set to 10:1. Subsequently, the processed data were converted to AutoGluon’s TabularDataset format, and the binary categorization model was constructed using TabularPredictor. We used 5-fold bagging to enhance the robustness of the model and a preset parameter “best_quality” to optimize the model performance. Taking the use of only H3K27ac signal as input as an example, the models generated by AutoGluon and their performance rankings on the test set are detailed in Table S4, available as supplementary data at *Bioinformatics* online.

The test set was predicted using AutoGluon’s TabularPredictor. We used all models trained by AutoGluon as base classifiers. The binary classification prediction results (“1” indicates the presence of enhancers, “0” means the absence of enhancers) output by each base classifier on the test set are used as new input features and fed into the meta-classifier (KAN) (Liu *et al.* 2024). Precisely, we stacked the predictions of each base classifier by columns, which ultimately constituted a new feature set. This feature set contains the prediction information from different base classifiers and was eventually merged with the feature sets of other signal data as the input to the meta-classifier of the second layer (Layer 2).

##### 2.1.2.2 *Layer 2 of Blending-KAN*

We merge the results of the first layer (Layer 1) into a new feature matrix to construct the inputs for the second layer (Layer 2). In the training phase, we use KAN as a meta-classifier. We employed a five-fold cross-validation strategy to ensure the reliability of the model evaluation. We use Stratified k-fold cross-validation to ensure that the distribution of category labels in each fold is consistent with the original data, thus avoiding the effect of category imbalance. During the training of each fold, we optimize the model using the Adam optimizer and train the network using the cross-entropy loss function. The model updates the parameters by mini-batch gradient descent. In the testing phase, the model performs inference on the test set in the no-gradient update mode and calculates the prediction accuracy. We also evaluate AUROC (area under the receiver operating characteristic curve) and AUPRC (area under the precision-recall curve) to fully reflect the model’s classification performance and ensure the accuracy and robustness of the evaluation results.

To validate the choice of KAN as the meta-classifier, we compared it with models like LightGBM, Logistic Regression, Naive Bayes, and MLP (see Text S5, available as supplementary data at *Bioinformatics* online).

### 2.2 Localization of the boundaries of enhancers

**Figure 3.**
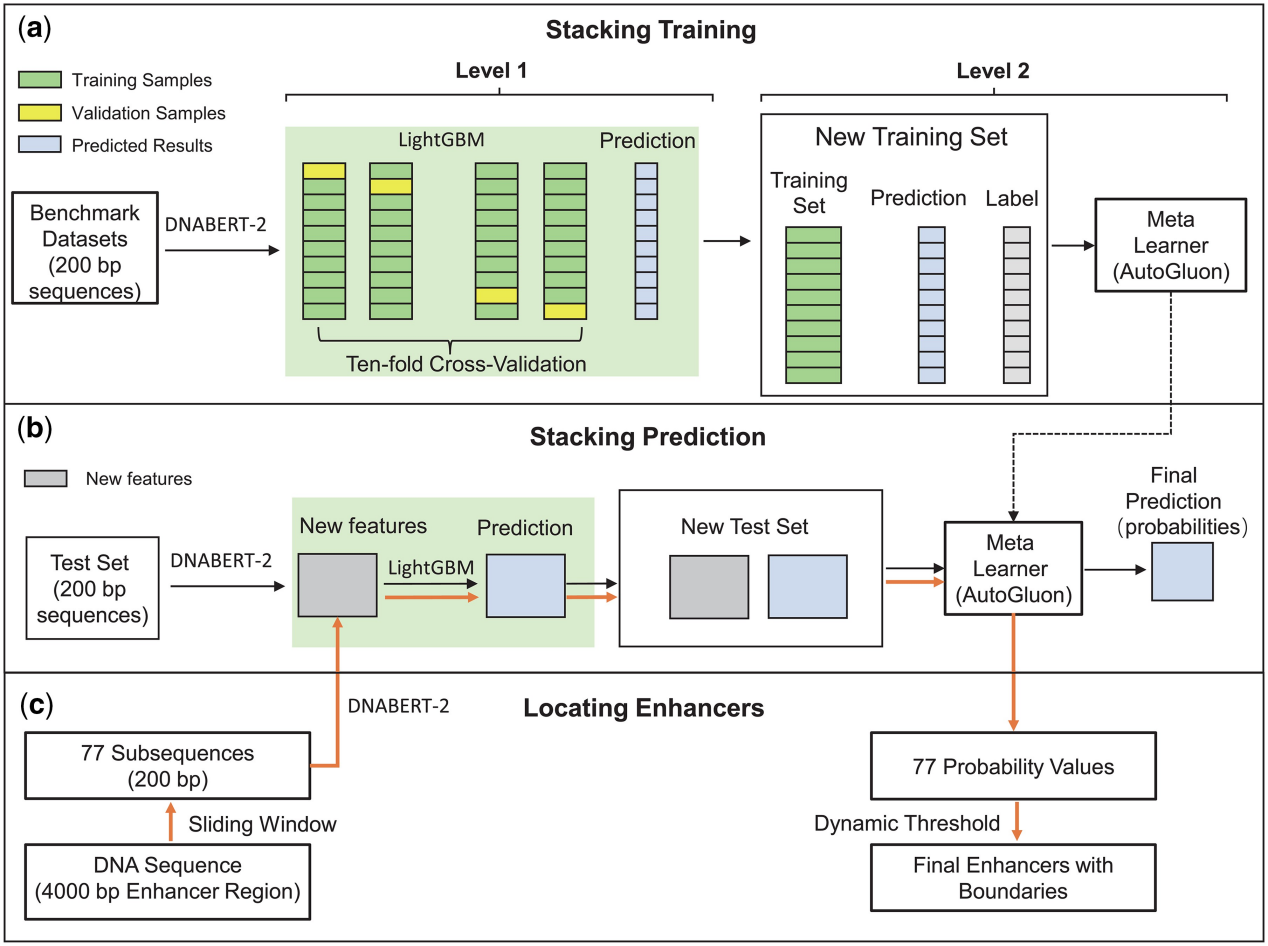
The architecture of the Stacking-Auto model. (a) Train the Stacking-Auto model. (b) Test the Stacking-Auto model. (c) Locating enhancers using the Stacking-Auto model.

We designed the Stacking-Auto model to localize the boundaries of enhancers from the regions containing enhancers (Fig. 3). First, we extracted the DNA sequences of the regions. Then, we split the sequences into 200 bp subsequences in a sliding window fashion (with a step size of 50 bp). Second, we designed the Stacking-Auto model to calculate the probability of a subsequence as an enhancer based on DNA sequences. We designed the Stacking-Auto model to get the probability that subsequences were enhancers. Finally, we developed a dynamic thresholding algorithm to determine the boundaries of enhancers based on the probabilities.

#### 2.2.1 Stacking-Auto model

To train a suitable model for determining the boundaries of enhancers, we used the benchmark datasets introduced in iEnhancer-2L (Liu *et al.* 2016) (see Text S6, available as supplementary data at *Bioinformatics* online and Fig. 2b and c for details of benchmark datasets). We input the datasets into DNABERT-2 (Zhou *et al.* 2023) to get embedded representations of samples (see Text S7, available as supplementary data at *Bioinformatics* online).

Stacking is an advanced integrated learning method that improves the generalization performance of a model by integrating the prediction results from different levels of learners. We used the LightGBM model (Li *et al.* 2018) as the base learner for the first layer (Level 1). LightGBM becomes our first choice for the first layer learner because of its ability to handle complex nonlinear relationships with high efficiency. We obtained the predictions of the first layer (Level 1) on the training set using ten-fold cross-validation. Then, we merged these predictions with the original input features to construct a new training set in Level 2. Subsequently, it was fed into a meta-learner to train a new final model.

While training the model in Level 1, we adopted the 10-fold cross-validation. The predictions of each fold were summarized into a new set of features called “meta-features”. After completing the 10-fold cross-validation, the meta-features of all the folds were combined into a complete feature set. These “meta-features” were stitched with the original features to form a richer and more comprehensive information representation. These expanded feature sets were used as inputs to the meta-learner. The meta-learner was also based on the AutoGluon framework, from which the best-performing model was selected as the meta-classifier. In the model training phase, we used AutoGluon’s TabularPredictor class to build the model and train it using the fit method. After training, we used the predict method to predict the independent test set and comprehensively evaluated the model performance.

#### 2.2.2 Locating enhancers

**Figure 4.**
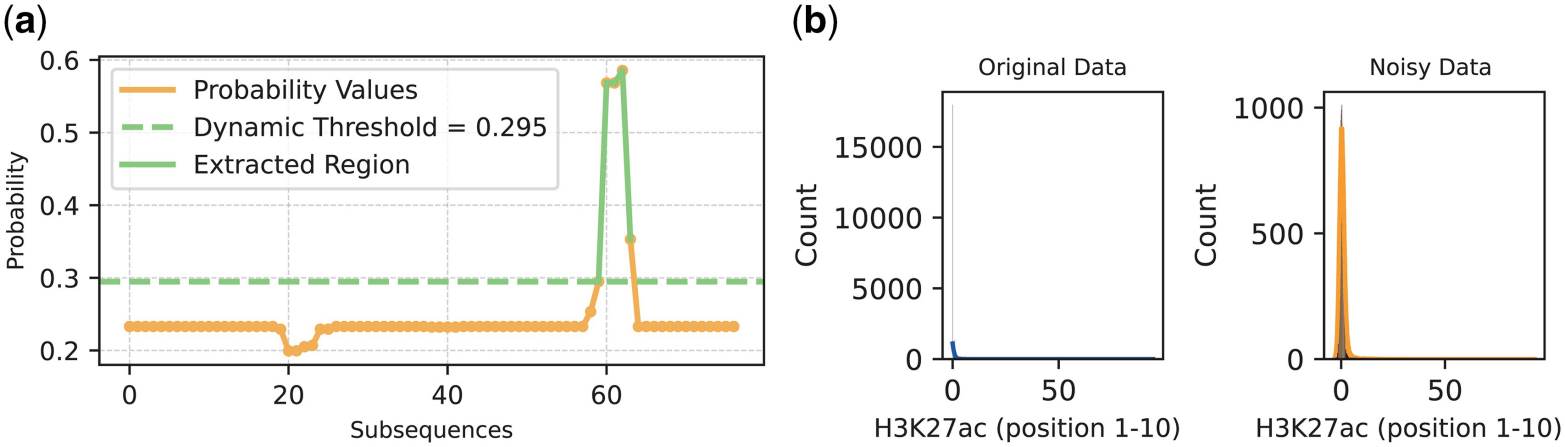
Probabilities of subsequences being enhancers and distribution of epigenetic signals. (a) The distribution of 77 probabilities of subsequences being enhancers. (b) Effect of Gaussian noise on the distribution of epigenetic signals (standard deviation σ = 0.9).

To pinpoint the boundaries of an enhancer, we segmented a 4000 bp enhancer region into 77 subsequences using sliding windows (see Text S8, available as supplementary data at *Bioinformatics* online and Fig. 2d) and used DNABERT-2 to extract embedding features (Fig. 3c). These embedding features were then processed by the Stacking-Auto model to generate the 77 probabilities of subsequences being enhancers. We designed a dynamic thresholding algorithm to pinpoint the boundaries of enhancers. The algorithm first calculates the mean and standard deviation of the probability values of all subsequences, and then sets the dynamic threshold as the mean minus 0.3 times the standard deviation. Subsequences with high probability are kept by this threshold, and neighboring retained subsequences are merged into a consecutive region. Ultimately, the region with the highest average probability is selected as the localization region of the enhancer. This method can adaptively adjust the threshold value to more accurately identify the boundaries of the enhancer (see Text S9, available as supplementary data at *Bioinformatics* online and Fig. 4a).

## 3 Results

### 3.1 Performance of Blending-KAN on various signal combinations

**Figure 5.**
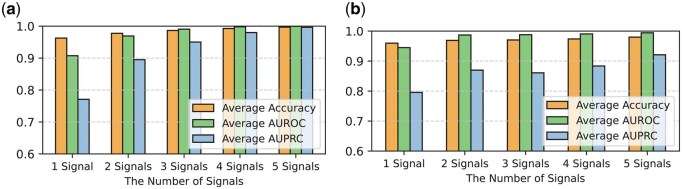
Performance evaluation. (a) Classification performance of Blending-KAN under different combinations of epigenetic signals. (b) Performance of Blending-KAN on cross-cell line prediction.

Compared to DECODE, the Blending-KAN model exhibits significant performance advantages (see Text S4, available as supplementary data at *Bioinformatics* online) and can support the free combination of various signals without relying on all five signals. In this section, we present the classification performance of Blending-KAN under various combinations of epigenetic signals. We evaluated the model’s performance using five-fold cross-validation and calculated the standard deviation for each evaluation metric. These metrics include accuracy, AUROC, AUPRC, and running time (in seconds) (Fig. 5a and Table S5, available as supplementary data at *Bioinformatics* online). These metrics and their standard deviations reflect the stability and superiority of Blending-KAN in handling diverse signal combinations.

Detailed performance metrics for each signal combination are provided in Table S5, available as supplementary data at *Bioinformatics* online. An analysis of the impact of the number of signal combinations on Blending-KAN is presented in Text S10, available as supplementary data at *Bioinformatics* online. As the number of combined signals increases, model performance continues to improve, especially when all five signals (DNase-seq, H3K27ac, H3K4me3, H3K4me1, and H3K9ac) are used, achieving the highest accuracy.

In summary, Blending-KAN performs excellent classification under various signal combinations, especially when combining DNase-seq and histone modification signals. By continuously increasing the variety of signals, Blending-KAN can effectively expand the feature space and thus improve the accuracy of enhancer detection. The flexibility of the model allows the flexible selection and combination of different signals to balance the cost of data acquisition and classification performance, providing an effective solution for the joint analysis of multiple signals. This characteristic has particular application in research environments where signal data acquisition is costly.

### 3.2 Robustness of the model to gaussian noise

To assess the robustness of Blending-KAN in the face of noise, we added varying degrees of Gaussian noise to the data (H3k27ac, H3k4me3, H3k9ac, H3K4me1, and DNase-seq), with the noise standard deviation (σ) ranging from 0.1 to 0.9, and the results were cross-validated in a five-fold. Fig. 4b shows the change in data distribution of the H3k27ac signal before and after adding noise. With the addition of noise, the data distribution widens, the variance increases, and the data points change from a centralized distribution to a wider spread. This change reflects the perturbing effect of noise on the data and is a common phenomenon when dealing with real observations (see Text S11, available as supplementary data at *Bioinformatics* online and Fig. 4b).

As shown in Table 1, the classification performance of Blending-KAN shows a certain decreasing trend after adding different levels of Gaussian noise in signals, especially in the high noise condition.

**Table 1.** Blending-KAN’s performance on data with Gaussian noise (mean ± standard deviation).

Noise (σ)	Mean accuracy	Mean AUROC	Mean AUPRC
0.1	0.9956 ± 0.0011	0.9992 ± 0.0004	0.9987 ± 0.014
0.3	0.9929 ± 0.0010	0.9983 ± 0.0005	0.9784 ± 0.0055
0.5	0.9934 ± 0.0012	0.9983 ± 0.0003	0.9757 ± 0.0071
0.7	0.9875 ± 0.0010	0.9952 ± 0.0013	0.9631 ± 0.0003
0.9	0.9874 ± 0.0003	0.9954 ± 0.0010	0.9635 ± 0.0076

Low noise level (σ = 0.1): the model performs best when the noise standard deviation is 0.1, with an accuracy of 0.9956, an AUROC of 0.9992, and an AUPRC of 0.9987. This indicates that Blending-KAN can stably classify augmented subregions at lower noise levels, maintaining high accuracy and discriminative power.

Medium noise level (σ = 0.3 and σ = 0.5): the performance of Blending-KAN decreases at noise standard deviations of 0.3 and 0.5. The accuracies are 0.9929 and 0.9934, respectively, and the AUROC and AUPRC also show a slight decrease. This suggests that the moderate noise level has some impact on the classification accuracy, especially in terms of AUPRC, where the model performance is degraded.

High noise level (σ = 0.7 and σ = 0.9): the performance of Blending-KAN decreases significantly as the noise standard deviation increases to 0.7 and 0.9. The accuracy drops to 0.9875 and 0.9874, the AUROC to 0.9952 and 0.9954, and the AUPRC to 0.9631 and 0.9635, respectively. Nevertheless, the model still maintains relatively high accuracy, indicating that Blending-KAN is still able to perform enhancer identification effectively in noisy situations, but has limited ability for higher noise adaptation ability is limited.

By adding different levels of Gaussian noise and performing five-fold cross-validation, the experimental results show that the Blending-KAN model can cope better with low to moderate noise levels and still maintain high classification performance. However, as the standard deviation of the noise increases, the model performance decreases accordingly, especially in the AUPRC metric. The significant decrease in AUPRC may indicate that the increase in noise reduces the model’s precision and recall in distinguishing between different classes.

### 3.3 Performance of Blending-KAN on cross-cell line prediction

To evaluate the performance of Blending-KAN in cross-cell line prediction, we trained the model using data from the HCT116 cell line and performed forecasts with data from the A549 cell line (Fig. 5b).

#### 3.3.1 Cross-cell line prediction using a kind of signal

Table S6, available as supplementary data at *Bioinformatics* online demonstrates the results of cross-cell line prediction using a kind of signal. The performance varied widely with a single signal for cross-cell line prediction. When using H3K27ac, the accuracy was 0.9566, AUROC was 0.9748, AUPRC was 0.8358, and the run time was 848 s. When using other single signals, the accuracies fluctuated between 0.9372 and 0.9700, suggesting that different epigenetic signals contribute differently to the transcellular lineage prediction.

#### 3.3.2 Cross-cell line prediction using two kinds of signals

The cross-cell line prediction performance of Blending-KAN was generally significantly improved on signal combinations. Table S7, available as supplementary data at *Bioinformatics* online demonstrates the cross-cell line prediction using two types of signals. When combining H3K27ac and H3K4me3, the accuracy reached 0.9676, the AUROC was 0.9830, and the AUPRC was 0.8587.

#### 3.3.3 Cross-cell line prediction using three or more kinds of signals

The prediction performance of Blending-KAN is further improved as the number of signal combinations increases. Table S8, available as supplementary data at *Bioinformatics* online demonstrates the results of cross-cell line prediction with multiple signal combinations, which shows a very high prediction accuracy of 0.9688, AUROC of 0.9924, and AUPRC of 0.9147 when trained with three signals (H3k27ac +H3k4me3 + DNase-seq). When using a combination of four signals (H3k27ac + H3k4me3 + H3k9ac + DNase-seq), the model achieved an accuracy of 0.9676, an AUROC of 0.9818, and an AUPRC of 0.7546, which is a slight decrease in performance but still better than a single signal.

#### 3.3.4 Best performance of the five kinds of signal combinations

Among all signal combinations, Blending-KAN trained with five signals performed the best, with an accuracy of 0.9798, an AUROC of 0.9942, and an AUPRC of 0.9209. Although the run time increased compared to the single signal and dual-signal combinations, the model’s performance in cross-cell line prediction was optimal thanks to the rich features provided by the five signals.

Experimental results of Blending-KAN in cross-cell line prediction show that the classification performance of the model is significantly improved with the increase in the number of signals. In particular, under the combination of five epigenetic signals, Blending-KAN can effectively fuse multiple signal information, improving the accuracy and stability of enhancer recognition.

### 3.4 Comparison of Stacking-Auto with existing methods

We also proposed a Stacking-Auto model that uses DNABERT-2 to extract features and AutoGluon as a base classifier and meta-classifier, aiming at locating enhancers. To evaluate the effectiveness of Stacking-Auto, we compared and analyzed it with 17 existing methods on an independent test set (see Text S6, available as supplementary data at *Bioinformatics* online), including iEnhancer-2L (Liu *et al.* 2016), EnhancerPred (Jia and He 2016), iEnhancer-DSNet (Asim *et al.* 2020), iEnhancer-Deep (Kamran *et al.* 2022), etc. The metrics are accuracy, sensitivity, specificity, and Matthews’s correlation coefficient (MCC). The results are shown in Table 2.

**Table 2.** Performance comparison of Stacking-Auto with 17 existing methods.

Methods^a^	Accuracy	Sensitivity	Specificity	MCC
iEnhancer-2L (Liu *et al.* 2016)	73.00	71.00	75.00	0.46
EnhancerPred (Jia and He 2016)	74.00	73.50	74.50	0.48
iEnhancer-DSNet (Asim *et al.* 2020)	78.00	78.00	77.00	0.56
iEnhancer-Deep (Kamran *et al.* 2022)	74.02	81.50	67.00	0.49
iEnhancer-EL (Liu *et al.* 2018)	74.45	71.00	78.50	0.50
Rank-GAN (Geng *et al.* 2022)	75.25	74.87	75.63	0.51
Le *et al.*’s BERT (Le *et al.* 2021)	75.60	80.00	71.20	0.51
iEnhancer-XG (Cai *et al.* 2021)	75.75	74.00	77.50	0.51
Tan *et al.* Enhancer (Tan *et al.* 2019)	76.00	76.00	76.00	0.51
iEnhancer-ECNN (Nguyen *et al.* 2019)	76.90	78.50	75.20	0.54
iEnhancer-EBLSTM (Niu *et al.* 2021)	77.20	75.50	79.50	0.53
iEnhancer-CNN (Khanal *et al.* 2020)	77.50	78.25	79.00	0.59
iEnhancer-DCLA (Liao *et al.* 2022)	78.25	78.00	78.50	0.57
iEnhancer-GAN (Yang *et al.* 2021)	78.40	81.10	75.80	0.57
Enhancer-RD (Yang *et al.* 2021)	78.80	81.00	76.50	0.58
iEnhancer-5Step (Le *et al.* 2019)	79.00	**82.00**	76.00	0.58
iEnhancer-MRBF (Xiao *et al.* 2022)	79.75	**82.00**	77.50	0.60
**Ours**	**80.50**	80.50	**80.50**	**0.61**

a Best results are marked in bold.

Stacking-Auto performs well in several metrics, especially regarding accuracy and MCC. Specifically, the accuracy of Stacking-Auto reaches 80.50%, which is slightly higher than the 79.75% of iEnhancer-MRBF (Xiao *et al.* 2022). Stacking-Auto’s sensitivity is 80.50%, which maintains a high accuracy while considering the higher specificity of 80.50%. Among all methods, 80.50% is the highest, showing a strong ability in negative sample recognition. Notably, Stacking-Auto achieves an MCC value of 0.61, the highest among all methods. This indicates that the model is better balanced in dealing with the class imbalance problem and can locate the augmented subsequence more stably.

Our Stacking-Auto model outperforms existing methods, mainly due to its significant advantages in feature extraction, automated learning, multi-model integration, and meta-classifier optimization. First, with DNABERT-2, we can extract deep DNA sequence features, which provide richer biological information for prediction and improve data quality. Meanwhile, the automation feature of AutoGluon allows the model to automatically complete feature engineering, model selection, and hyperparameter tuning, significantly reducing human intervention and improving generalization ability. In addition, Stacking-Auto employs the Stacking strategy to integrate the prediction results of base classifiers and enhance the overall prediction performance through meta-classifier optimization integration. Selected meta-classifiers further optimize these integration results, enabling Stacking-Auto to demonstrate higher accuracy and stability in the augmented subsequence localization task.

## 4 Discussion

This study proposes a new framework for enhancer prediction and localization based on Blending-KAN and Stacking-Auto models. First, enhancer and non-enhancer regions are predicted on multidimensional epigenetic signals using Blending-KAN. Enhancer regions are segmented into 200 bp subsequences by a sliding window, and Stacking-Auto further predicts the probability of each subsequence being an enhancer. Ultimately, based on these probability values, a dynamic thresholding algorithm is used to determine the boundaries of enhancers accurately. The accuracy of our framework outperforms existing methods.

### 4.1 The advantages of Blending-KAN in enhancer prediction

The Blending-KAN model efficiently predicts enhancer regions by integrating different epigenetic signals, combining the weighted fusion of multi-base classifiers and the nonlinear mapping advantage of KAN. Experimental results show that the introduction of multiple signal combinations (e.g. DNase-seq and H3K4me1) significantly improves the accuracy and stability of the classification. The KAN model is able to further explore the nonlinear relationship between different signals and enhance the recognition of complex regulatory regions. In addition, the weighted fusion method performs well in solving the category imbalance problem, enabling the model to maintain stable predictions under different noise levels. Compared with methods that rely on fixed signal combinations (e.g. DECODE), Blending-KAN’s flexibility enables it to achieve excellent performance even when different signals are available. Meanwhile, automated feature engineering, model selection, and hyperparameter tuning based on the AutoGluon framework further enhance the model’s efficiency and performance.

### 4.2 A novel strategy for enhancer localization

For enhancer localization, we used a sliding window strategy to partition a 4000 bp sequence into 200 bp overlapping subsequences and calculated the probability of each subsequence being an enhancer using Stacking-Auto. Stacking-Auto effectively captures sequence features in different subsequences through DNABERT-2 feature extraction, AutoGluon, and Stacking. Through the dynamic thresholding algorithm of probability mean and standard deviation, we flexibly extracted the high-probability regions and merged the neighboring segments to determine the complete enhancers. This method is highly adaptable to different samples and effectively improves the accuracy of enhancer localization.

## Supplementary Material

btaf441_Supplementary_Data

## Data Availability

The data underlying this article are available at https://github.com/emanlee/Hi-Enhancer and https://doi.org/10.6084/m9.figshare.29262158.v1.
