# Supplementary material for: Hi-Enhancer: a two-stage framework for prediction and localization of enhancers based on Blending-KAN and Stacking-Auto models
Source: Bioinformatics. 2025 Dec 8;42(1):btaf441. doi: 10.1093/bioinformatics/btaf441 (PMC12758598; doi:10.1093/bioinformatics/btaf441)
Supplement: btaf441_Supplementary_Data [file btaf441_supplementary_data.docx]

**Hi-Enhancer: a two-stage framework for prediction and localization of enhancers based on Blending-KAN and Stacking-Auto models**

Content

[Text S1. Succinct description of models and techniques. 2](#_Toc203169576)

[Text S2. Rationale and reasons for cell line selection. 3](#_Toc203169577)

[Text S3. Optimizing sample ratio for enhancer region detection. 3](#_Toc203169578)

[Text S4. Dataset division and comparison with DECODE. 4](#_Toc203169579)

[Text S5. Meta-classifier Selection 4](#_Toc203169580)

[Text S6. Benchmark datasets and performance evaluation metrics. 4](#_Toc203169581)

[Text S7. Extracting sequence features using DNABERT-2. 5](#_Toc203169582)

[Text S8. Sliding window to extract subsequences. 6](#_Toc203169583)

[Text S9. A dynamic thresholding algorithm to pinpoint the boundaries of enhancers. 6](#_Toc203169584)

[Text S10. Analysis of the effect of different signal combinations on the performance of Blending-KAN. 7](#_Toc203169585)

[Text S11. Adding Gaussian noise to the signal data. 8](#_Toc203169586)

[Table S1. Blending-KAN dataset overview. 8](#_Toc203169587)

[Table S2. Performance comparison of different meta-classifiers. 8](#_Toc203169588)

[Table S3. Hyperparameter settings for the KAN model. 9](#_Toc203169589)

[Table S4. Performance of base classifiers based on H3k27ac and AutoGluon. 9](#_Toc203169590)

[Table S5. Classification performance of Blending-KAN under different combinations of epigenetic signals. 9](#_Toc203169591)

[Table S6. Performance of Blending-KAN in cross-cell line prediction using a kind of signal. 10](#_Toc203169592)

[Table S7. Performance of Blending-KAN in cross-cell line prediction using two kinds of signals. 11](#_Toc203169593)

[Table S8. Performance of Blending-KAN in cross-cell line prediction of multiple signal combinations. 11](#_Toc203169594)

Text S1. Succinct description of models and techniques.

Blending is an integrated learning method that involves dividing the dataset into a training set and a validation set. First, multiple base classifiers are trained on the training set. Then, the prediction results of these classifiers on the validation set are used as new features to reconstruct the training set. Finally, a meta-classifier is trained to integrate these new features. This approach can fully utilize the advantages of different base classifiers and improve the overall performance of the model through multi-level feature fusion. In the first stage of the Hi-Enhancer framework (Blending-KAN model), Blending is used to train multiple base classifiers on the training set and generate new features for the meta-classifier. This method helps in effectively combining the predictions of different classifiers to improve the accuracy of enhancer region prediction.

KAN (Kolmogorov-Arnold Network) is a neural network architecture based on the Kolmogorov-Arnold super-definite approximation theorem, which can decompose a complex multivariate function into a series of univariate function combinations, thus realizing the effective modeling of complex nonlinear relationships. This network architecture can capture complex nonlinear features in the data with a shallow number of network layers, avoiding the overfitting problem that traditional deep neural networks may encounter. In the Blending-KAN model, KAN acts as a meta-classifier, integrating the prediction results of the base classifiers and capturing complex nonlinear relationships in the data, thereby significantly improving the accuracy of enhanced subregion predictions. KAN receives the probability values output by multiple base classifiers as input features and, relying on its unique network structure and optimization algorithm, generates the final enhanced subregion prediction results.

AutoGluon is an automated machine learning (AutoML) framework that automates a series of machine learning tasks such as data preprocessing, feature engineering, model selection and hyperparameter tuning. It finds the optimal model and hyperparameter combination for a given dataset by integrating multiple machine learning algorithms and automated search strategies to improve model performance and generalization. In the first stage of the Blending-KAN model, we use the AutoGluon framework to train multiple base classifiers. AutoGluon automates the feature engineering and model selection processes to ensure that the base classifiers achieve optimal performance and generalization. Specifically, the task of the base classifiers is to preliminarily classify genomic regions based on input epigenetic signals (DNase-seq, H3K27ac, etc.) to determine whether the region is likely to contain an enhancer. AutoGluon can automatically handle tasks such as data preprocessing, feature extraction, model selection, and hyperparameter tuning, training the optimal base classifier for each epigenetic signal. These base classifiers preliminarily classify genomic regions based on their respective input signals, determine whether the region may contain an enhancer, and output corresponding probability values. These probability values are integrated into the meta-classifier KAN as new features for further comprehensive judgment.

Stacking is an advanced integrated learning approach that improves the overall prediction performance by combining models at different levels and utilizing a meta-learner to weight or fuse the predictions of these models. This approach can make full use of the advantages of each base model to improve the model's ability to handle complex problems through multi-level feature fusion and model optimization. In the second stage of the Hi-Enhancer framework (Stacking-Auto model), Stacking is used to combine the predictions from different base models and optimize the final prediction of enhancer boundaries. This method helps in improving the accuracy and robustness of enhancer localization.

DNABERT-2 is a DNA sequence language model based on the Transformer architecture, which converts DNA sequences into a series of tokens by Byte Pair Encoding (BPE) technology, and then encodes these tokens using the Transformer layer to generate embedded representations of each token. These embedded representations contain rich DNA sequence feature information and can capture both local and global features of the sequence. DNABERT-2 is used in the Stacking-Auto model to extract deep features from DNA sequences. The embedded representations generated by DNABERT-2 serve as the input features for the Stacking model, enabling it to accurately predict the probability of each subsequence being an enhancer and ultimately localize the enhancer boundaries.

Text S2. Rationale and reasons for cell line selection.

In this study, we selected two cell lines, HCT116, a human colon cancer cell line widely used to study the molecular mechanisms and gene regulatory networks of colorectal cancer, and A549, a human lung adenocarcinoma cell line commonly used to study the molecular mechanisms and gene regulation of lung cancer. Both cell lines have high-quality and complete epigenetic modification data in the ENCODE program, including chromatin accessibility (DNase-seq) and histone modification (ChIP-seq), which provide reliable training and validation resources for enhancer prediction. By performing cross-cell line predictions between the HCT116 and A549 cell lines, we could assess the generalization ability and robustness of the model to ensure its applicability across different cell types. In addition, these two cell lines represent colorectal cancer and lung cancer, respectively, two very common cancer types with important research value and representativeness.

Our reasons for selecting only these two cell lines were mainly based on research aims and resource constraints. Our goal was to develop a flexible and high-performance framework that not only performs well with a single signal or a combination of signals but also maintains high accuracy in both individual cell line training and cross-cell line prediction. This means that even if a user has only a kind of signal data from one cell line, he or she can still train effectively with our model. To this end, we not only provide pre-trained models but also support users to select specific cell lines and chromatin markers for customized model training according to their needs. The training process, code, and video tutorials are publicly available on GitHub, allowing users to flexibly adjust the input signals to better meet their research goals. Although the ENCODE project covers data resources from a wide range of cell lines, data integrity and quality vary across cell lines. To ensure the accuracy and reliability of our models during the training process, we prioritized cell lines with high-quality data and rich epigenetic modification data reserves.

Text S3. Optimizing sample ratio for enhancer region detection.

In constructing the model, we chose a ratio of 1:10 between positive samples (enhancer regions) and negative samples (non-enhancer regions.) This ratio was chosen based on the following considerations: first, the number of enhancer regions in the actual genomic data is much less than that of non-enhancer regions, and this imbalance needs to be reflected in model training; second, through experimental validation, we found that the ratio of 1:10 can enable the model to achieve a better balance between accuracy and generalization ability; finally, the choice of this ratio also takes into account the reasonable use of computational resources.

Text S4. Dataset division and comparison with DECODE.

In the Blending algorithm, dividing the dataset is a very crucial step because the effectiveness of Blending depends heavily on how the training data is divided. To determine the most appropriate division ratio in this study, we used all five signals and tried five division ratios (5:5, 6:4, 7:3, 8:2, and 9:1), and experiments were conducted on the Blending-KAN model. At the same time, training was performed on DECODE using the same dataset. The results are shown in Figure 2a, where our model exhibits the best performance under the 7:3 division ratio. This indicates that under this division strategy, the training of base classifiers and the generation of meta-classifiers can effectively synergize to maximize the advantages of each model, thus improving the overall prediction ability.

It is worth noting that the Blending-KAN method outperforms DECODE at different training and testing set partition ratios, especially at the 7:3 ratio. Under this ratio, Blending-KAN's accuracy, AUROC, and AUPRC reach 0.9969, 0.9997, and 0.9963, respectively, all of which are significantly better than DECODE's.

Text S5. Meta-classifier Selection

To verify the rationality of choosing KAN as a meta-classifier, we selected LightGBM, Logistic Regression, Naive Bayes, and MLP as meta-classifiers and trained them with the five signals as inputs. In the model evaluation process, we adopt a five-fold cross-validation strategy and calculate the three key metrics of Accuracy, AUROC, and AUPRC on the testing set for these models, as shown in Table S2.

Based on the evaluation results, the KAN model performed best across all three metrics and demonstrated superior overall performance compared to other models. However, the computational efficiency of KAN is slightly lower than that of MLP, particularly when handling large-scale datasets, where standard MLP may offer advantages in terms of GPU utilization efficiency and scalability. Nevertheless, the dataset used in this study is large in scale; the data used for training the meta-classifier accounts for only approximately 30% of the total dataset, and its input feature dimension is significantly lower than that of the base classifier. Therefore, the additional computational overhead introduced by KAN is within an acceptable range during actual training. Considering both model performance and training efficiency, we ultimately selected KAN as the meta-classifier. The hyperparameter settings for the KAN model are shown in Table S3.

Text S6. Benchmark datasets and performance evaluation metrics.

To train a suitable model for determining the boundaries of enhancers, we used the benchmark dataset introduced in iEnhancer-2L [[1](#_ENREF_1)] . This dataset contains enhancer sequences collected from nine different cell lines, which were divided into 200 bp segments. In addition, highly similar DNA sequences (>20% similarity) were removed using CDHIT [[2](#_ENREF_2)] . The final benchmark dataset includes 1484 enhancers (742 weak and 742 strong enhancers) and 1484 non-enhancers, as well as 200 enhancers (100 weak and 100 strong enhancers) and 200 non-enhancers in the independent test set, as shown in Figure 2b-c. We uniformly regard strong and weak enhancers as positive samples, and this dataset has been widely used in studies related to the classification of DNA enhancers.

When evaluating a model on an independent test set, the Stacking-Auto model outputs a probability value for each subsequence that is used to indicate the likelihood that the subsequence is an enhancer. To convert these probability values into specific prediction categories (enhancers or non-enhancers), subsequences with probability values greater than or equal to 0.5 are determined to be in the positive category (enhancers), while subsequences with probability values less than 0.5 are determined to be in the negative category (non-enhancers). This method is simple and intuitive, and is widely used in binary classification problems.

To comprehensively evaluate the performance of the Stacking-Auto model in the enhancer boundary determination task, we use four metrics: Accuracy, AUROC, AUPRC, and MCC.

$Accuracy =(TP+TN)/ (TP+TN+FN+FP)$ (1)

where TP (True Positive) denotes the number of subsequences correctly predicted as enhancers; TN (True Negative) denotes the number of subsequences correctly predicted as non-enhancers; FP (False Positive) denotes the number of subsequences incorrectly predicted as enhancers; and FN (False Negative) denotes the number of incorrectly predicted non-enhancer number of subsequences.

The AUROC value reflects the model's overall ability to distinguish between enhancers and non-enhancers. A higher AUROC value indicates that the model can more effectively differentiate between enhancers and non-enhancers across different thresholds. We calculate the AUROC using the probability values output by the model, by assessing the changes in True Positive Rate (TPR) and False Positive Rate (FPR).

AUPRC measures the Precision and Recall of the model at different thresholds. Precision is the proportion of subsequences correctly predicted as enhancers over all subsequences predicted as enhancers, and Recall is the proportion of subsequences correctly predicted as enhancers over all actual enhancers. Similarly, we calculate the area under the curve (AUPRC) using the probability values output by the model.

MCC is a balanced metric that integrates true examples, false positive examples, true negative examples, and false negative examples. The value of MCC ranges from -1 to 1, with values closer to 1 indicating better model performance. The calculation formula is as follows:

$MCC=$/$\sqrt{(TP+FP)(TP+FN)(TN+FP)(TN+FN)}$ (2)

The overall performance of the Stacking-Auto model performed consistently with no significant differences in the two cell lines: HCT116 and A549.

Text S7. Extracting sequence features using DNABERT-2.

Before locating enhancers, we input the above samples into DNABERT-2 [[3](#_ENREF_3), [4](#_ENREF_4)]. This model employs the Byte Pair Encoding (BPE) technique to transform the input DNA sequences into a series of tokens, and each token goes through a series of Transformer layers, which ultimately outputs an embedded representation of each token. Among these outputs, we pay special attention to the final hidden states of the sequences, and these representations contain rich sequence feature information. Next, we use these features as inputs for downstream model training.

Text S8. Sliding window to extract subsequences.

To precisely localize enhancers, the regions containing enhancers were segmented using the sliding window method. As shown in Figure 2d, the length of the sliding window is 200 bp, and the step size is 50 bp. Each 4000 bp sequence was divided into 77 subsequences. There are 50 bp overlapping regions between these subsequences to reduce the likelihood of potential enhancer regions being missed. In subsequent steps, the 200 bp subsequences were used as independent samples for feature extraction and obtaining the probability of active enhancers.

Text S9. A dynamic thresholding algorithm to pinpoint the boundaries of enhancers.

To pinpoint the boundaries of the enhancers, we segmented a 4000 bp DNA sequence of an enhancer into 77 subsequences and used DNABERT-2 to extract embedding features. These embedding features were then processed by the Stacking-Auto model to generate the 77 probabilities of subsequences being enhancers. To better understand the distribution of these probabilities, we have drawn a graph (Figure 4a) showing the distribution of probability values for each subsequence in a sample. The dashed line indicates the dynamic threshold for this sample, and the solid green line indicates the final enhancer.

To determine the boundaries of enhancers, we set a dynamic threshold for each sample, defined as the mean of all probability values minus 0.3 times the standard deviation. This dynamic threshold can be expressed by the following Equation (3).

$Threshold=\mu-0.3\sigma$ (3)

where the mean $\mu$ and standard deviation $\sigma$ are calculated as Equations (4) and (5).

$\mu=\frac{1}{n}\sum_{i=1}^{n} P_{i}$ (4)

$\sigma=\sqrt{\frac{1}{n-1}\sum_{i=1}^{n} {{(P}_{i}-\mu)}^{2}}$ (5)

where $P_{i}$ denotes the probability value of the $i-th$ subsequence and$n$ is the total number of subsequences (77 in this case). This dynamic threshold setting effectively captures relatively high probability subsequence fragments, ensuring that we identify regions with high enhancer potential.

Based on this filtering criterion, we merged neighboring high-probability segments to form consecutive regions. For each merged region, we calculate its average probability. Among all the candidate regions, we selected the region with the highest average probability as the final region of the enhancer. Finally, we calculated the start and end coordinates of the region in the genome based on its relative position in the original 4000 bp sequence. The start and end coordinates were calculated by Equations (6) and (7):

$Start Coordinate=Original Start Position+start\times20$ (6)

$End Coordinate=Original Start Position+(end+1)\times200-1$ (7)

where *Original Start Position* is the start coordinate of the 4000 bp sequence in the genome, *start* and *end* are the start and end subsequence indexes of the merged region, respectively.

Text S10. Analysis of the effect of different signal combinations on the performance of Blending-KAN.

(1) Manifestation of a single signal

The performance of Blending-KAN differed significantly when using one of five signals. Blending-KAN demonstrated optimal classification performance when using DNase-seq signals alone. Specifically, an accuracy of 0.9958 was achieved, with an AUROC of 0.9994 and an AUPRC of 0.9934. This result highlights the importance of chromatin accessibility in enhancer identification, demonstrating its strength in capturing sequence features. In contrast, single signals using histone modification (H3K27ac, H3K4me3, etc.) were slightly less impressive in performance, illustrating the limitations of single histone modification information in identifying complex biological functional regions. Run time for the single signal typically ranged from 4500 to 5200 seconds, suggesting that it is computationally efficient and particularly suitable for rapid initial screening.

(2) Advantages of the dual-signal combination

When using a two-signal combination, the performance of Blending-KAN is generally improved, especially in AUROC and AUPRC. For example, the combination of H3K4me1 with DNase-seq achieved an accuracy of 0.9961, with an AUROC of 0.9996 and an AUPRC of 0.9942. This result indicates that combining chromatin accessibility with histone modification signals can better capture the features of gene regulatory regions. Although the run time is about 9000 seconds, the performance improvement is significant, proving the advantage of multi-signal fusion.

(3) Boosting of three-signal combinations

When further increasing the number of signals to three, the overall performance of the model continued to improve, especially in terms of accuracy and AUPRC. For example, the combination of H3K9ac, H3K4me1, and DNase-seq achieved an accuracy of 0.9962, with an AUROC and AUPRC of 0.9996 and 0.9959, respectively. This suggests that an increase in the number of signal types helps the model to capture a richer set of features, which in turn improves the classification accuracy. The run time of the three-signal combination is approximately between 13500 and 14500 seconds, and its performance improvement is certainly worthwhile despite the increased computational cost.

(4) Balance of four-signal combinations

In the case of four-signal combinations, the overall performance of Blending-KAN is close to that of five-signal combinations. For example, the combination of H3K27ac, H3K9ac, H3K4me1, and DNase-seq achieves an accuracy of 0.9967, an AUROC of 0.9996, and an AUPRC of 0.9946. This combination achieves a good balance between the feature space and the model complexity. Still, at the same time, the AUPRC of this combination is lower than the three-signal combination of H3K4me3, H3K4me1, and DNase-seq, which indicates that the signal combination should be flexibly selected according to the specific needs in practical applications.

(5) Optimal performance of five-signal combinations

Under the five-signal combination condition, Blending-KAN achieves the optimal classification performance. The accuracy of this combination is 0.9969, AUROC is 0.9997, and AUPRC is 0.9963, indicating that the combined use of the five signals can maximize the feature information in the data and optimize the accuracy of enhancer detection. Although the run time grows to 23062 seconds, the enriched feature space allows Blending-KAN to optimize the performance in enhancer recognition.

Text S11. Adding Gaussian noise to the signal data.

To assess the robustness of Blending-KAN in the face of noise, we added varying degrees of Gaussian noise to the data (H3k27ac, H3k4me3, H3k9ac, H3K4me1, and DNase-seq), with the noise standard deviation ($\sigma$) ranging from 0.1 to 0.9, and the results were cross-validated in a five-fold. Specifically, the addition of noise followed the following Equation (8):

$s'=s+\epsilon, \epsilon\sim N(0,\sigma^{2})$ (8)

where $s'$ is the signal after noise addition, $s$ is the original signal, $\epsilon$ is the noise drawn from a standard normal distribution, and the standard deviation of the noise$(\sigma$) controls the amplitude of the noise.

Figure 4b illustrates the data distribution of the H3k27ac signal without or with noise (taking the first column of data as an example). Specifically, adding noise resulted in an extension of the data distribution with a slight shift in the signal's center. At the same time, the variance of the data has increased, reflecting that the noise has perturbed the data. The original data distribution is very centralized, while the data distribution shows a broader bell-shaped curve after adding noise. This indicates that the addition of noise has caused an increase in data variability; the data points are no longer concentrated but are distributed over a broader range. This variation is common in data analysis, especially when encountering actual observations where noise is unavoidable.

Table S1. Blending-KAN dataset overview.

| Name | Source | Cell lines | Positive samples | Negative samples |
| --- | --- | --- | --- | --- |
| DNase-seq | ENCODE | HCT116, A549 | 8350 | 83500 |
| H3K27ac | ENCODE | HCT116, A549 | 8350 | 83500 |
| H3K4me3 | ENCODE | HCT116, A549 | 8350 | 83500 |
| H3K4me1 | ENCODE | HCT116, A549 | 8350 | 83500 |
| H3K9ac | ENCODE | HCT116, A549 | 8350 | 83500 |

Table S2. Performance comparison of different meta-classifiers.

| Models | Mean Accuracy | Mean AUROC | Mean AUPRC |
| --- | --- | --- | --- |
| LightGBM | 0.9927±0.0009 | 0.9989±0.0004 | 0.9845±0.0073 |
| Logistic Regression | 0.9930±0.0013 | 0.9988±0.0005 | 0.9843±0.0081 |
| Naive Bayes | 0.9848±0.0030 | 0.9918±0.0014 | 0.8599±0.0209 |
| MLP | 0.9935±0.0003 | 0.9990±0.0062 | 0.9869±0.0062 |
| KAN | **0.9969± 0.0011** | **0.9997± 0.0003** | **0.9963 ± 0.0025** |

Note: mean ± standard deviation.

Table S3. Hyperparameter settings for the KAN model.

| Parameter name | Parameter values |
| --- | --- |
| Layers_hidden | [400,128,64] |
| Grid_size | 5 |
| Spline_order | 3 |
| Scale_noise | 0.1 |
| Scale_base | 1.0 |
| Scale_spline | 1.0 |
| Base_activation | torch.nn.SiLU |
| Grid_eps | 0.02 |
| Grid_range | [-1, 1] |
| Epochs | 10 |
| Batch_size | 32 |

Table S4. Performance of base classifiers based on H3k27ac and AutoGluon.

| Model | Test  accuracy | Val  accuracy | Test time (seconds) | Val time  (seconds) | Training time  (seconds) |
| --- | --- | --- | --- | --- | --- |
| NeuralNetTorch_BAG_L2 | 0.9419 | 0.9866 | 17.9662 | 197.7721 | 1543.0636 |
| ExtraTreesEntr_BAG_L2 | 0.9418 | 0.9899 | 14.2157 | 208.0380 | 1440.1202 |
| NeuralNetFastAI_BAG_L2 | 0.9414 | 0.9930 | 16.9679 | 190.6560 | 1639.3894 |
| LightGBM_BAG_L2 | 0.9411 | 0.9944 | 14.1322 | 187.5913 | 1464.0949 |
| ExtraTreesGini_BAG_L2 | 0.9411 | 0.9908 | 14.2376 | 208.2060 | 1440.0850 |
| XGBoost_BAG_L2 | 0.9407 | 0.9946 | 15.0092 | 189.8726 | 1501.6035 |
| CatBoost_BAG_L2 | 0.9406 | 0.9931 | 14.0856 | 186.6938 | 1571.8243 |
| WeightedEnsemble_L3 | 0.9405 | 0.9947 | 15.1898 | 191.1346 | 1554.1454 |
| LightGBMLarge_BAG_L2 | 0.9404 | 0.9947 | 14.1105 | 187.4744 | 1448.0507 |
| RandomForestEntr_BAG_L2 | 0.9394 | 0.9918 | 14.1433 | 208.4884 | 1461.3446 |
| RandomForestGini_BAG_L2 | 0.9391 | 0.9926 | 14.2026 | 208.0145 | 1458.7172 |
| LightGBMXT_BAG_L2 | 0.9371 | 0.9846 | 14.0966 | 187.4308 | 1444.6667 |
| LightGBMXT_BAG_L1 | 0.9347 | 0.9558 | 0.9091 | 23.8863 | 1198.7124 |
| WeightEnsemble_L2 | 0.9286 | 0.9658 | 7.2820 | 104.1300 | 1213.8290 |
| LightGBM_BAG_L1 | 0.9197 | 0.9089 | 0.3390 | 1.8064 | 225.9580 |
| KNeighborsDist_BAG_L1 | 0.8387 | 0.9102 | 6.3691 | 80.0190 | 2.6827 |
| KNeighborsUnif_BAG_L1 | 0.8386 | 0.9102 | 6.3160 | 80.6831 | 2.7327 |

Table S5. Classification performance of Blending-KAN under different combinations of epigenetic signals.

| Signals | Mean Accuracy | Mean AUROC | Mean AUPRC | Run Time |
| --- | --- | --- | --- | --- |
| H3k27ac | 0.9429 ± 0.0028 | 0.8145 ± 0.0149 | 0.5845 ± 0.0222 | **4594** |
| H3k4me3 | 0.9499 ± 0.0039 | 0.8333 ± 0.0150 | 0.6611 ± 0.0363 | 4909 |
| H3k9ac | 0.9545 ± 0.0019 | 0.8988 ± 0.0103 | 0.7303 ± 0.0236 | 4719 |
| H3K4me1 | 0.9707 ± 0.0026 | 0.9709 ± 0.0069 | 0.8504 ± 0.0322 | 4691 |
| DNase-seq | **0.9958 ± 0.0006** | **0.9994 ± 0.0003** | **0.9934 ± 0.0008** | 5137 |
| H3k27ac+H3k4me3 | 0.9550 ± 0.0030 | 0.8931 ± 0.0082 | 0.7410 ± 0.0210 | 9239 |
| H3k27ac+H3k9ac | 0.9552 ± 0.0028 | 0.9079 ± 0.0100 | 0.7654 ± 0.0201 | 9028 |
| H3k27ac+H3K4me1 | 0.9722 ± 0.0015 | 0.9872 ± 0.0026 | 0.8986 ± 0.0172 | **9023** |
| H3k27ac+DNase-seq | 0.9955 ± 0.0012 | 0.9994 ± 0.0003 | 0.9942 ± 0.0011 | 9464 |
| H3k4me3+H3k9ac | 0.9589 ± 0.0026 | 0.9303 ± 0.0089 | 0.7808 ± 0.0243 | 9345 |
| H3k4me3+H3K4me1 | 0.9733 ± 0.0028 | 0.9867 ± 0.0018 | 0.9081 ± 0.0105 | 9326 |
| H3k4me3+DNase-seq | 0.9955 ± 0.0003 | 0.9993 ± 0.0003 | 0.9925 ± 0.0019 | 9754 |
| H3k9ac+H3K4me1 | 0.9735 ± 0.0014 | 0.9867 ± 0.0028 | 0.8826 ± 0.0270 | 9145 |
| H3k9ac+DNase-seq | 0.9956 ± 0.0008 | 0.9993 ± 0.0004 | 0.9934 ± 0.0020 | 9527 |
| H3K4me1+DNase-seq | **0.9961 ± 0.0003** | **0.9996 ± 0.0001** | **0.9942 ± 0.0016** | 9560 |
| H3k27ac+H3k4me3+H3k9ac | 0.9594 ± 0.0025 | 0.9337 ± 0.0085 | 0.8051 ± 0.0194 | 13699 |
| H3k27ac+H3k4me3+H3K4me1 | 0.9751 ± 0.0017 | 0.9894 ± 0.0017 | 0.9151 ± 0.0137 | 13689 |
| H3k27ac+H3k4me3+DNase-seq | 0.9952 ± 0.0015 | 0.9994 ± 0.0002 | 0.9938 ± 0.0020 | 14117 |
| H3k27ac+H3k9ac+H3K4me1 | 0.9754 ± 0.0015 | 0.9890 ± 0.0014 | 0.9151 ± 0.0120 | **13641** |
| H3k27ac+H3k9ac+DNase-seq | 0.9956 ± 0.0004 | 0.9993 ± 0.0003 | 0.9933 ± 0.0020 | 13939 |
| H3k27ac+H3K4me1+DNase-seq | 0.9948 ± 0.0018 | 0.9995 ± 0.0003 | 0.9941 ± 0.0031 | 13927 |
| H3k4me3+H3k9ac+H3K4me1 | 0.9756 ± 0.0015 | 0.9892 ± 0.0007 | 0.9052 ± 0.0200 | 13840 |
| H3k4me3+H3k9ac+DNase-seq | 0.9959 ± 0.0008 | 0.9994 ± 0.0003 | 0.9953 ± 0.0014 | 14276 |
| H3k4me3+H3K4me1+DNase-seq | 0.9959 ± 0.0003 | **0.9996 ± 0.0001** | 0.9946 ± 0.0020 | 14263 |
| H3k9ac+H3K4me1+DNase-seq | **0.9962 ± 0.0005** | **0.9996 ± 0.0002** | **0.9959 ± 0.0018** | 14041 |
| H3k27ac+H3k4me3+H3k9ac+H3K4me1 | 0.9783 ± 0.0022 | 0.9910 ± 0.0010 | 0.9252 ± 0.0152 | 18154 |
| H3k27ac+H3k9ac+H3K4me1+DNase-seq | **0.9967 ± 0.0013** | 0.9996 ± 0.0003 | 0.9946 ± 0.0045 | 18399 |
| H3k27ac+H3k4me3+H3K4me1+DNase-seq | 0.9960 ± 0.0007 | 0.9995 ± 0.0003 | 0.9949 ± 0.0032 | 18619 |
| H3k27ac+H3k4me3+H3k9ac+DNase-seq | 0.9960 ± 0.0011 | 0.9994 ± 0.0004 | 0.9957 ± 0.0013 | 18653 |
| H3k4me3+H3k9ac+H3K4me1+DNase-seq | 0.9966 ± 0.0010 | **0.9997 ± 0.0002** | **0.9958 ± 0.0023** | 18685 |
| H3k27ac+H3k4me3+H3k9ac+H3K4me1+DNase-seq | **0.9969 ± 0.0011** | **0.9997 ± 0.0003** | **0.9963 ± 0.0025** | **23062** |

**Note: Bold text indicates the best results (mean ± standard deviation).**

Table S6. Performance of Blending-KAN in cross-cell line prediction using a kind of signal.

| Signal | Accuracy | AUROC | AUPRC | Run time |
| --- | --- | --- | --- | --- |
| H3k27ac | 0.9566 | 0.9748 | 0.8358 | **848** |
| H3k4me3 | 0.9661 | 0.9417 | 0.8145 | 1618 |
| H3k9ac | **0.9700** | 0.9806 | 0.8683 | 1192 |
| H3K4me1 | 0.9372 | 0.8330 | 0.5367 | 1186 |
| DNase-seq | 0.9687 | **0.9940** | **0.9223** | 2256 |

**Note: Bold text indicates the best results.**

Table S7. Performance of Blending-KAN in cross-cell line prediction using two kinds of signals.

| Two signals | Accuracy | AUROC | AUPRC | Run time |
| --- | --- | --- | --- | --- |
| H3k27ac+H3k4me3 | 0.9676 | 0.9830 | 0.8587 | 2418 |
| H3k27ac+H3k9ac | 0.9695 | 0.9854 | 0.8603 | 2148 |
| H3k27ac+H3K4me1 | 0.9625 | 0.9800 | 0.8138 | 2185 |
| H3k27ac+DNase-seq | 0.9690 | 0.9942 | 0.9344 | 2791 |
| H3k4me3+H3k9ac | 0.9726 | 0.9796 | 0.8409 | 2477 |
| H3k4me3+H3K4me1 | 0.9661 | 0.9811 | 0.8201 | 2084 |
| H3k4me3+DNase-seq | 0.9675 | 0.9930 | 0.9234 | 2442 |
| H3k9ac+H3K4me1 | 0.9580 | 0.9833 | 0.7875 | **1803** |
| H3k9ac+DNase-seq | 0.9720 | 0.9932 | 0.9008 | 2212 |
| H3K4me1+DNase-seq | **0.9846** | **0.9959** | **0.9574** | 2233 |

**Note: Bold text indicates the best results.**

Table S8. Performance of Blending-KAN in cross-cell line prediction of multiple signal combinations.

| Three to five signals | Accuracy | AUROC | AUPRC | Run time |
| --- | --- | --- | --- | --- |
| H3k27ac+H3k4me3+H3k9ac | 0.9602 | 0.9712 | 0.7950 | 2786 |
| H3k27ac+H3k4me3+H3K4me1 | 0.9713 | 0.9889 | 0.8779 | 2763 |
| H3k27ac+H3k4me3+DNase-seq | 0.9688 | 0.9924 | 0.9147 | 3185 |
| H3k27ac+H3k9ac+H3K4me1 | 0.9656 | 0.9849 | 0.8152 | **2546** |
| H3k27ac+H3k9ac+DNase-seq | 0.9706 | 0.9880 | 0.8016 | 2981 |
| H3k27ac+H3K4me1+DNase-seq | **0.9790** | **0.9961** | **0.9532** | 2961 |
| H3k4me3+H3k9ac+H3K4me1 | 0.9714 | 0.9837 | 0.7969 | 2955 |
| H3k4me3+H3k9ac+DNase-seq | 0.9639 | 0.9875 | 0.8266 | 3354 |
| H3k4me3+H3K4me1+DNase-seq | 0.9755 | 0.9940 | 0.9322 | 3376 |
| H3k9ac+H3K4me1+DNase-seq | 0.9771 | 0.9922 | 0.8944 | 3130 |
| H3k27ac+H3k4me3+H3k9ac+H3K4me1 | 0.9694 | 0.9890 | 0.8751 | **3686** |
| H3k27ac+H3k9ac+H3K4me1+DNase-seq | 0.9763 | **0.9942** | **0.9338** | 3873 |
| H3k27ac+H3k4me3+H3K4me1+DNase-seq | **0.9780** | 0.9940 | 0.9318 | 4086. |
| H3k27ac+H3k4me3+H3k9ac+DNase-seq | 0.9676 | 0.9818 | 0.7546 | 4084 |
| H3k4me3+H3k9ac+H3K4me1+DNase-seq | 0.9774 | 0.9935 | 0.9227 | 4253 |
| H3k27ac+H3k4me3+H3k9ac+H3K4me1+DNase-seq | **0.9798** | **0.9942** | 0.9209 | **5022** |

**Note: Bold text indicates the best results.**

**References**

1. Liu B, Fang L, Long R *et al*: **iEnhancer-2L: a two-layer predictor for identifying enhancers and their strength by pseudo k-tuple nucleotide composition**. *Bioinformatics* 2016, **32**(3):362-369.

2. Fu L, Niu B, Zhu Z *et al*: **CD-HIT: accelerated for clustering the next-generation sequencing data**. *Bioinformatics* 2012, **28**(23):3150-3152.

3. Zhou Z, Ji Y, Li W *et al*: **Dnabert-2: Efficient foundation model and benchmark for multi-species genome**. *arXiv preprint arXiv:230615006* 2023.

4. Sennrich R: **Neural machine translation of rare words with subword units**. *arXiv preprint arXiv:150807909* 2015.
